# BUILDING RESILIENCE AND GRIT IN HIGHER EDUCATION’S OWN CLIMATE CRISIS

**DOI:** 10.1093/geroni/igae098.0838

**Published:** 2024-12-31

**Authors:** David Burdick, Christine Ferri

**Affiliations:** Stockton University, Galloway, New Jersey, United States; Stockton University, Galloway, New Jersey, United States

## Abstract

Recently, the climate (& emotional ‘temperature’) and context for gerontology in higher education has never been a more difficult predicament. We will either be collateral damage from enrollment-based budget cuts, eroding societal respect for the value of a degree, and/or culture-war conflicts against DEI initiatives, or we could position ourselves to be embraced (by administration & others) as part of the solution (e.g., through advocacy of age-inclusive initiatives that may diversify enrollment and funding streams). This paper considers strengths, weaknesses, opportunities, and perils for our work. After a brief discussion of the fraught macro/societal/secular context, we describe within our institutional context the 15-year experiences of the Stockton Center on Successful Aging (SCOSA). Founded in 2007 as a provost priority, seeking a “major center on aging,” an 8-person part-time staff now utilizes diverse funding streams to fulfill our research, education, and service mission. A changing institutional context has included administrative turnover (two deans, six provosts, and two presidents) and changing institutional priorities. With a wider lens, we’ll reassess past AGHE efforts of our lead author related to center/program survival in tough economic times (e.g., “Finding Funding” ‘99 & “What Works for Your Program/Department/Center” ‘10, AGHExchange columns, 2010 AGHE Reno Annual Meeting Pre-Conference Intensive with Gugliucci, and 3-year AGHE/GSA funding from the Retirement Research Foundation assisting AGHE to support programs & centers). As our first author passes the leadership torch to our second author upon his retirement after 40 years, we discuss the generational shift many other aging centers are also experiencing.

